# A five-domain functional framework for integrative physiology: a coherent and operational approach to whole-body function

**DOI:** 10.3389/fphys.2026.1771189

**Published:** 2026-06-12

**Authors:** Serena Y. Kuang

**Affiliations:** Department of Foundational Medical Studies, Oakland University William Beaumont School of Medicine, Rochester, MI, United States

**Keywords:** coherence, functional domain, integrative physiology, interdisciplinary research, internal environment, mathematical relationship, physiology education

## Abstract

Because the body is commonly described in a six-layered structural hierarchy starting from molecules and cells to tissues, organs, organ systems, and finally to the whole organism, bodily functions (physiological and pathophysiological) are often approached in the same layered way. This is familiar and convenient, but it has clear limitations. Many important functions, such as homeostasis, thermoregulation, stress responses, and so on, emerge from coordinated causal processes that span multiple levels. They cannot be understood by assigning them to a single anatomical layer, yet integrative physiology still lacks a functional framework that is both conceptually coherent and practically useful for representing such multi-level interactions. This article introduces a 5-domain functional framework, developed through logical analysis of how intracellular fluid compartment(s) (ICF or ICFs) operate collectively within the shared extracellular fluid compartment (ECF). From this enveloping ECF–ICF relationship, five relational functional domains emerge. This perspective organizes bodily processes according to their *operational logic* rather than their anatomical position, allowing multi-level coordination to be visualized in a unified and flexible way. This framework and logic further lead to the development of a second functional platform that can organize bodily functions described by quantitative/mathematical relationships (e.g., oxygen-hemoglobin dissociation curve, Nernst equation, Michaelis-Menten equation, etc.). The platform is named Blueprint for Quantitative Functional Organization (BQFO). When mathematical expressions of function are placed into the BQFO, they form a systematically organized hierarchy rather than remaining scattered across topics. Together, the 5-domain functional framework and the BQFO provide not only practical tools for integrating complex functions but also a more realistic epistemology and methodology for understanding how bodily functions operate. These developments potentially establish new principles and offer new tools for integrative physiology and interdisciplinary teaching and research.

## 1 Introduction

The organization of the human body is commonly described in a layered, structure-based manner, starting from molecules and cells to tissues, organs, organ systems, and finally the whole organism (traditional 6-level structural hierarchy). Bodily functions (physiological and pathophysiological) are often approached in the same layered way. This approach is convenient, but it has clear limitations. For example, many essential functions, such as blood pressure regulation, thermoregulation, fluid balance, or the stress response, do not reside within any single anatomical level but emerge from coordinated causal interactions that involve multiple cell types and different anatomical structures and mechanisms operating together over time.

Emergent properties are central to integrative physiology. The all-or-none behavior of an action potential results from the organized interplay of channel gating, membrane properties, and ionic gradients. Body temperature regulation requires the cooperation of peripheral sensors, hypothalamic integration, autonomic and endocrine systems, and behavior. Even health itself—an organism’s ability to maintain stability, adapt, and function—emerges from interactions among many systems rather than from any particular structural layer. A functional way of organizing processes that reflects how they operate together, not merely where their anatomical substrates reside, will strengthen our understanding of these phenomena. Nevertheless, although the need for a cross-level perspective is widely recognized, integrative physiology still lacks a functional framework that is both conceptually coherent and methodologically practical. The present work aims to address this need by proposing a 5-domain functional framework designed specifically for integrative physiology, with the goal of clarifying how distributed cellular and organ-level processes can be organized within a coherent representation of whole-body function.

The 5-domain functional framework organizes bodily functions according to their *operational logic* of roles, interactions, and causal flows rather than according to anatomical layers. Section 2 explains how the framework is developed through logical analysis.

In Section 3, the framework is applied to homeostasis, allostasis, and pathophysiological transitions to illustrate how distributed bodily processes can be represented visually within one coherent functional space. In other words, the functional logic revealed in Section 2 is instantiated in concrete, typical physiological and pathophysiological processes to show the effectiveness of the framework. The 5-domain framework also enables the development of a second functional tool. Expressing bodily functions mathematically represents a deeper level of understanding as it describes how variables change in relation to one another. However, these quantitative relationships are typically scattered across subjects without a unifying logic. Once this functional logic is established in the 5-domain framework, it is feasible to place many familiar quantitative relationships into a systematic context. This extension of the framework is presented in Section 4 as the Blueprint for Quantitative Functional Organization (BQFO), a platform/tool for organizing diverse mathematical relationships in a functional hierarchical order.

In Section 5, the significance and implications of the 5-domain functional framework are discussed.

The 5-domain functional framework and the BQFO offer a new epistemological perspective and a methodological approach to address the gap mentioned above. The broader significance and implications of the 5-domain framework and the BQFO are explored in Section 5.

## 2 Development of the 5-domain functional framework

The development of the framework involves two simple steps. First, the functional relationship between the extracellular fluid compartment (ECF) and the intracellular fluid compartment (ICF) must be properly conceptualized. Second, the functional domains are partitioned based on this relationship.

### 2.1 The relationship between the ECF and ICF

In physiology textbooks, the two conceptual/virtual body fluid compartments—ECF and ICF—are drawn side-by-side as two neighbor “compartments.” This presentation is convenient for teaching volumes and ionic compositions, but it overlooks the essential interdependence of the ECF and ICF. In reality, they are coupled through the cell membrane, which serves as the interface for the exchange of matter, energy, and information between the ECF and ICF. Treating them as neighbored entities obscures the relationship that makes physiological integration possible.

**Figure 1:**
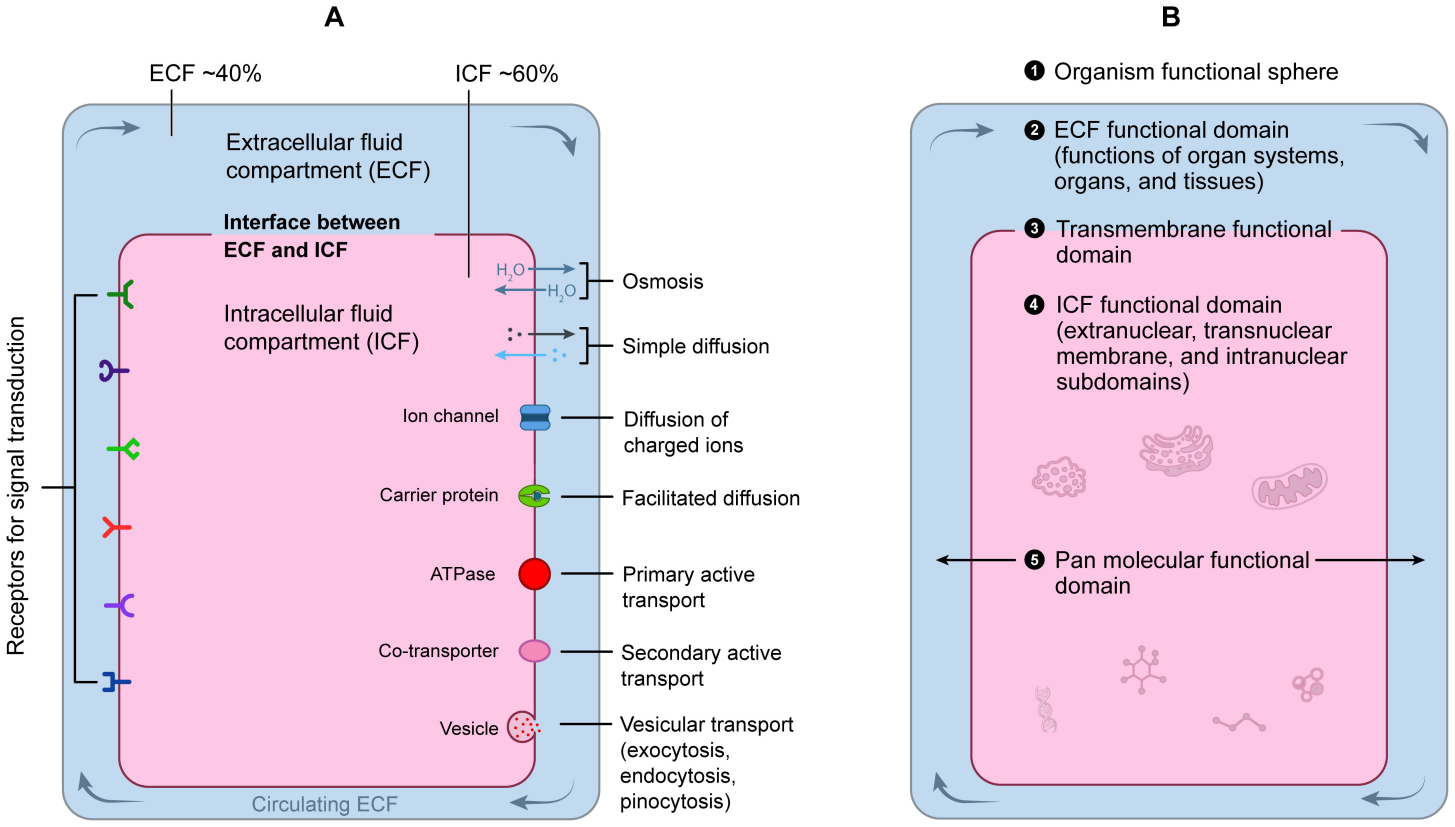
The 5-domain functional framework for integrative physiology. **(A)** ECF enveloping ICF. Rectangles schematically represent the approximate 60% ICF and 40% ECF volume distribution. The boundary between them forms a critical interface that enables various types of membrane transport mechanisms (passive and active) and receptor-mediated signal transduction. The circulating bulk ECF (the internal environment) ensures functional coherence among all cells, tissues, organs, and organ systems within it. The ICF maintains coherence inside each cell. **(B)** Partition of functional domains. Domain ❸ (transmembrane domain) is the interface between the ECF and ICF, where cross-boundary causation occurs. Inside it lies the intracellular functional domain (Domain ❹), which can be subdivided into the extranuclear, trans-nuclear membrane, and intranuclear subdomains (not shown). Surrounding this interface is the extracellular domain (Domain ❷), covering the functions of tissues, organs, and organ systems. Domain ❶ represents organism-level functions. Molecular events are ubiquitous across all domains. When needed, these may be conceptualized as a pan-molecular domain (Domain ❺). Intracellular receptors (e.g., steroid hormone receptors) participate in molecular signaling processes and are therefore represented within the intracellular domain (not shown).

In my previous article (Kuang, 2023), I illustrated a more functionally meaningful relationship: the ECF envelops the ICF because all cells are bathed in the internal environment, i.e., the ECF. This means that tissues, organs, and organ systems—being composed of cells—also operate within the ECF. **Figure 1A** adapts that earlier idea and clarifies the functional boundary between the ECF and ICF. This encapsulating relationship is abstract, but it shifts the usual static comparison of ECF versus ICF volumes into a relational and integrative view. Although schematic, it more closely reflects physiological reality. Its flexibility and explanatory value will be apparent in later examples.

### 2.2 Partitioning functional domains based on the ECF–ICF relationship

**Figure 1B** shows the resulting functional-domain partition derived from this enveloping relationship. The boundary between the ECF and ICF becomes the transmembrane functional domain (Domain ❸), which is essential for functional coherence but absent from the traditional 6-level structural hierarchy.

The partition shown in **Figure 1** represents a conceptual organization of functional domains derived from the encapsulating ECF–ICF relationship. This representation is intentionally abstract and is designed to clarify the underlying relational logic of physiological organization rather than to depict specific physiological systems. At this highest level of abstraction, the functional domains (particularly the transmembrane domain, i.e., Domain ❸)—are defined conceptually, emphasizing their roles in mediating the exchange of matter, energy, and information between the ECF and ICF.

Accordingly, at this highest level of abstraction (**Figure 1**), the transmembrane domain is not characterized by specific geometrical or structural parameters, such as membrane surface area. However, in applications of the framework to particular physiological processes, such geometrical factors may become relevant, as they can influence transport capacity and exchange efficiency. In these contexts, membrane surface area and related structural features can be incorporated as part of the context-dependent representation of the system without altering the underlying relational logic of the framework. In Section 3, when the abstract relational logic illustrated in **Figure 1** is applied to specific physiological processes, the ECF–ICF relationship is progressively instantiated in a context-dependent manner. At this level, Domain ❸ may incorporate additional structural or geometrical parameters, such as membrane surface area, when these factors are relevant to transport capacity or exchange efficiency in the system being represented.

Several additional distinctions follow naturally from this conceptual partition:

- The tissue, organ, and organ-system levels are grouped into the extracellular domain (Domain ❷) as their functions are simultaneously shaped by the bulk, circulating ECF.
- Correspondingly, the intracellular domain (Domain ❹) represents processes occurring within cells.
- The traditional cellular level in the 6-level hierarchy does not appear as an independent level; instead, it is functionally decomposed into extracellular-facing, transmembrane, and intracellular aspects.
- A potential extension, not pursued here, is to treat the skin as an additional functional domain at the organism–environment interface, which may become relevant when connecting internal physiology to broader organism–environment relationships in future work.

To apply the framework, the intracellular domains must be adapted to the context. Different cell types express different sets of transmembrane proteins; in principle, an organism with ~200 cell types could be represented with 200 distinct ICFs, each with its own unique transmembrane domain and ICF, but in practice, only the functionally relevant ICFs need to be depicted. It will become clear shortly, depending on the research or teaching purpose, that ICFs can be grouped, separated, or simplified to reveal the functional interplay among the selected cell types.

The next section provides examples that illustrate how this flexibility supports the representation of coordinated functions across levels. Although the framework contains five domains, only the first four are used in the examples. The pan-molecular domain is always present implicitly, as molecular determinants are embedded within the ECF, each ICF, and its transmembrane processes. Depending on the context and purpose of representation, molecular processes may remain backgrounded or become explicitly emphasized within the framework.

## 3 Application of the 5-domain framework

This section presents three examples to illustrate how the 5-domain framework organizes bodily processes under different conditions (physiological and pathophysiological) into coherent, cross-level representations. Each example shows how the distributed cell types interact through a shared extracellular environment and how their transmembrane and intracellular processes can be placed into one functional context. These illustrations are of familiar processes, so they can easily demonstrate how the framework clarifies relationships that are already well understood but often described in fragmented ways.

A fourth example is presented separately in Section 4 to extend the framework to quantitative relationships and demonstrate how the same logic can organize equations and curves commonly used in physiology and other life sciences.

### 3.1 Example 1: plasma osmoregulation, a classic homeostatic process

**Figure 2:**
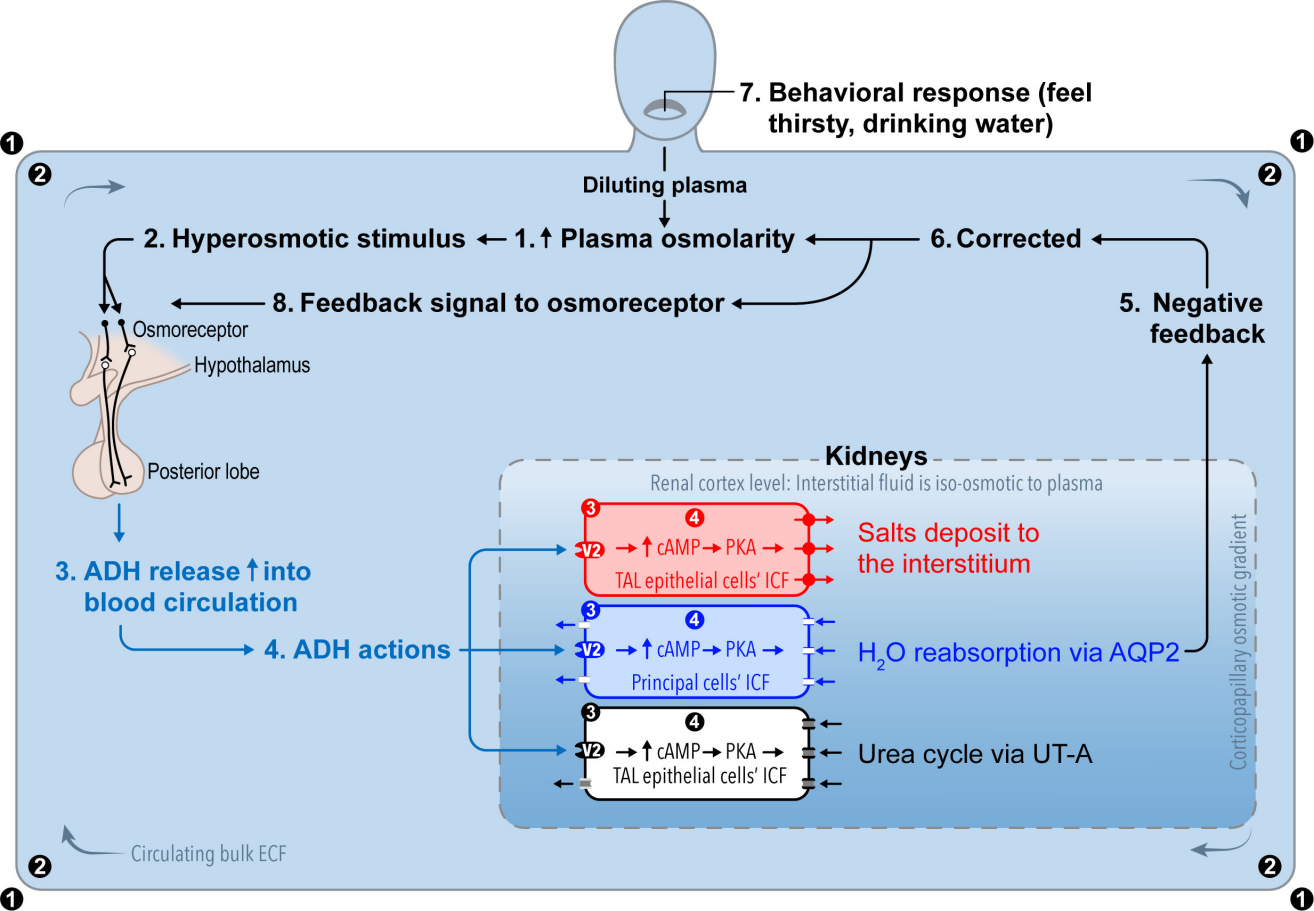
Application of the 5-domain functional framework to illustrate plasma osmoregulation. Labels ❶–❹ denote the first four functional domains. Domain ❷ represents the ECF, the internal environment in which all components operate. Its blue background indicates a healthy ECF condition. Within the transmembrane functional domain (❸), three representative renal epithelial cell types are shown, each highlighted in a distinct color (red, blue, and black) to indicate their unique sets of membrane transporters and signaling mechanisms. Their corresponding intracellular domains (❹) are delineated in matching colors, emphasizing that different cell types contribute differently to the overall regulatory loop. Directional arrows mark causal and temporal relationships across domains. Together with the spatial markers, they present a coherent sequence linking hypothalamic osmosensing, posterior pituitary secretion of ADH, renal epithelial responses restoring plasma osmotic concentration (OC), and organism-level behavioral response. For clarity, the hypothalamus and posterior pituitary are drawn directly within the ECF domain rather than being fully decomposed into their own domains (e.g., Domains ❸ and ❹ of osmosensory neurons or neurosecretory terminals). This selective simplification reduces visual complexity and demonstrates the flexible use of the framework: additional intracellular and transmembrane details can be added when needed or shown in supplementary diagrams. A corticopapillary osmotic gradient is included in the renal medulla as an example of a localized ECF niche embedded within the larger ECF domain. Although not required for every schematic, this feature illustrates how the framework accommodates local, osmotic gradient and spatial heterogeneity without losing coherence at the whole-system level. The pan-molecular domain (Domain ❺) is not shown separately but is implicitly embedded across all domains through the molecular mechanisms underlying signaling, membrane transport, and intracellular regulation. Schematic is not to scale.

As an initial illustration, plasma osmoregulation via ADH release is a classic example of homeostasis. The 5-domain functional framework provides a natural way to place these distributed elements into a single causal sequence. The shared healthy ECF environment (Domain ❷, blue) and the distinct transmembrane mechanisms (Domain ❸) of various renal epithelial cell types form the organizational backbone of the illustration in **Figure 2**. Their intracellular domains (❹) respond according to these mechanisms, and the arrows trace how changes propagate across domains.

Simplifications such as grouping hypothalamic and pituitary elements within the ECF domain emphasize that the framework allows intracellular detail to be expanded or collapsed without disrupting causal continuity. In this way, the same regulatory loop can be viewed at different levels of abstraction while preserving its integrated logic.

### 3.2 Example 2: systemic responses to hypoxia at high altitude, an allostatic regulation

**Figure 3:**
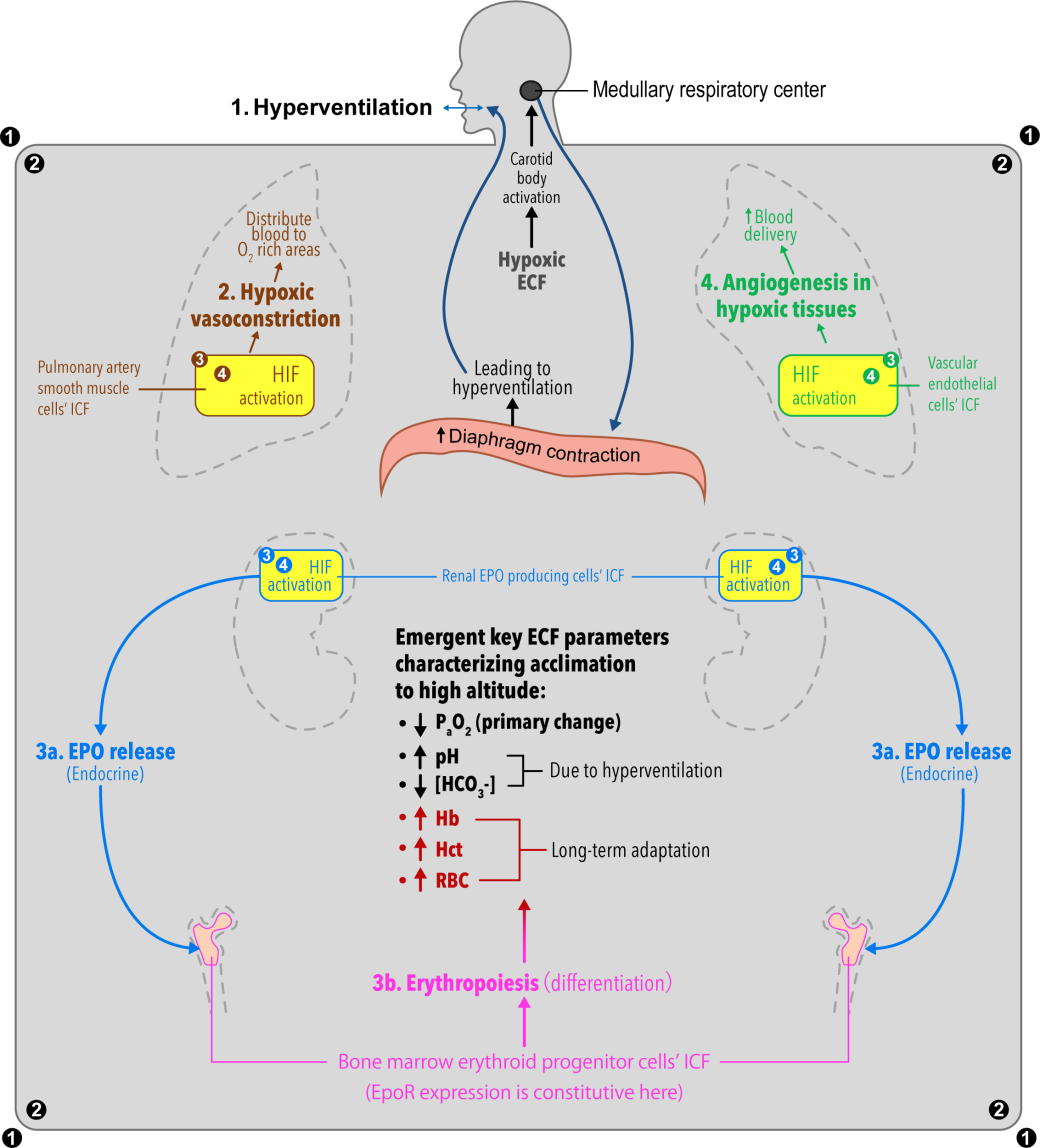
Application of the 5-domain functional framework to illustrate major allostatic responses to high-altitude hypoxia. In contrast to the healthy blue ECF domain (❷) in **Figure 2**, the gray background here represents a hypoxic internal environment, indicating that all cell types are operating within a shared oxygen-deprived ECF. Four major responses are presented in a simplified but coherent sequence. (1) Acute hyperventilation: Peripheral chemoreceptors detect acute hypoxia and activate brainstem respiratory centers, increasing ventilation through the coordinated activation of respiratory muscles (only the diaphragm is shown in the schematic). (2) Acute hypoxic pulmonary vasoconstriction: Pulmonary arterial smooth muscle cells constrict in poorly ventilated regions, redistributing blood flow and improving ventilation–perfusion matching. (3) Erythropoietin (EPO)-mediated erythropoiesis: Sustained hypoxia activates hypoxia-inducible factor (HIF) signaling in renal EPO-producing cells, increasing circulating EPO and stimulating erythropoiesis in the bone marrow. (4) Angiogenesis: Chronic hypoxia induces HIF-dependent signaling in endothelial cells, promoting vascular remodeling and increased capillary density in hypoxic tissues. In the schematic, this process is illustrated in the lung for visual clarity, although similar angiogenic responses may occur in multiple hypoxic tissues throughout the body. For visual clarity, hypoxic pulmonary vasoconstriction is illustrated in one lung and angiogenesis in the contralateral lung. In reality, both processes occur in both lungs, but the separation in the diagram helps emphasize their conceptual distinction while avoiding visual overcrowding. Color coding highlights the different intracellular domains (❹) participating in these responses: bright yellow ICFs represent hypoxia-sensitive cells responding directly to the hypoxic ECF, while the pink bone-marrow ICFs represent erythroid progenitors responding indirectly to circulating EPO. Carotid body glomus cells are shown as hypoxia-sensitive, but their intracellular pathways are intentionally not expanded, demonstrating the selective abstraction allowed by the framework. Schematic is not to scale.

Acclimatization to high altitude is an allostatic process in which several heterogeneous, acute and chronic responses take places. The 5-domain framework provides a coherent structure to organize these different responses (**Figure 3**).

Selective simplifications are intentionally used in **Figure 3** to demonstrate the flexibility of the framework. The ghosted lines of organs (lungs and kidneys) deemphasize anatomical detail to draw attention to the functional domains involved and facilitate teaching and learning. Depending on the purpose, the representation can be adjusted by increasing or reducing the level of abstraction. For example, the organ outlines could be removed and identical intracellular (ICF) regions merged into a single compartment, producing a more abstract and simplified depiction that is better suited for theoretical analysis while also creating space to elaborate selected intracellular signaling processes. The framework could also be further systematized in future developments by consolidating equivalent intracellular compartments, expanding the representation of relevant intracellular mechanisms, and developing a series of coordinated diagrams to illustrate different stages of the allostatic process, from the initial hypoxic perturbation to the emergence of a new steady physiological state after acclimatization. Overall, this example illustrates that the value of the framework lies not in depicting every mechanistic detail, but in providing a coherent functional logic for organizing distributed physiological responses.

### 3.3 Example 3: metabolic overload and insulin resistance, a transition from homeostasis to allostatic failure

**Figure 4:**
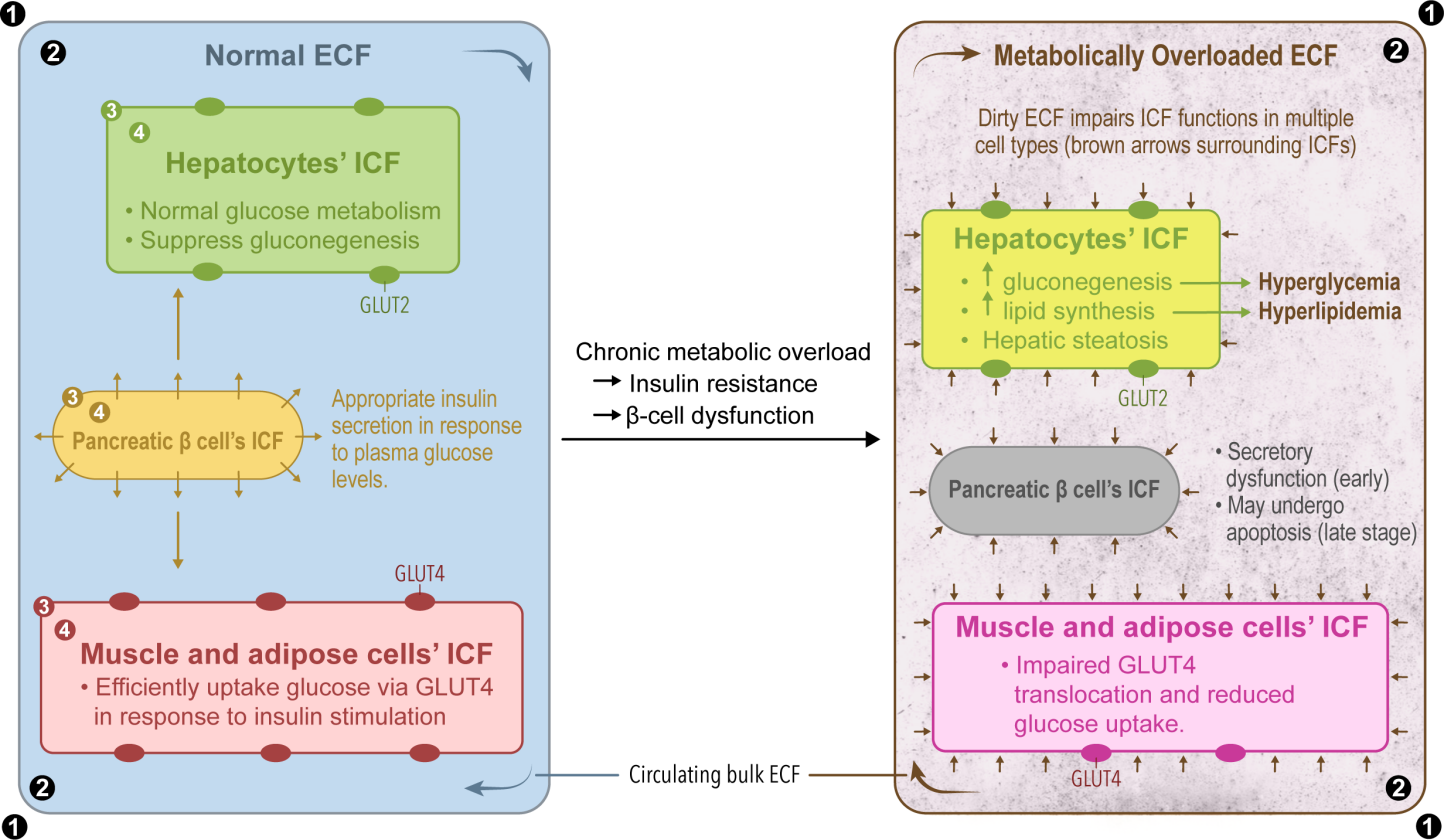
Application of the 5-domain functional framework to illustrate the transition from normal insulin-regulated glucose metabolism to insulin resistance. On the left, a healthy ECF domain (❷, blue background) supports coordinated actions among hepatocytes, pancreatic β cells, and insulin-responsive muscle and adipose cells. Their intracellular domains (❹) function normally, and their transmembrane domains (❸) mediate appropriate glucose uptake (via GLUT4), glucose sensing, and suppression of hepatic gluconeogenesis. On the right, chronic metabolic overload transforms the ECF into a maladaptive internal environment (brown, clouded background). This altered ECF drives diverse intracellular disturbances across multiple cell types indicated using multiple short brown arrows surrounding each type of ICF. Hepatocyte ICFs (yellow-green) show increased gluconeogenesis, lipid synthesis, and features of hepatic steatosis. Muscle and adipose ICFs (pink) exhibit impaired GLUT4 translocation, reducing insulin-stimulated glucose uptake. Pancreatic β-cell ICFs (gray) display secretory dysfunction and may undergo apoptosis in later stages. These disturbances in Domain ❹, mediated through altered transmembrane signaling (Domain ❸), collectively generate systemic hyperglycemia and hyperlipidemia.

**Figure 4** illustrates how the 5-domain framework can also represent the breakdown of coordinated function. Here, chronic metabolic overload alters the ECF environment and drives diverse intracellular disturbances across several cell types.

A key feature of the diagram is the merging of muscle and adipose intracellular domains into a single ICF compartment. In the context of impaired GLUT4 translocation, both cell types show essentially the same functional response. When physiological questions require distinguishing their divergent roles, their ICFs can be represented separately. This illustrates the purpose-driven flexibility of the 5-domain framework: ICF compartments may be merged or separated depending on the level of coordinated function that the illustration aims to convey.

### 3.4 Integrative insights and scalability of the framework

These three examples show how the 5-domain functional framework provides a spatially integrated and causally continuous way to depict bodily processes under different conditions (physiological and pathophysiological). By nesting all intracellular domains within a shared extracellular environment, the framework makes the transmembrane domain (❸) emerge naturally as the functional interface between the ECF and ICF. This organization is not readily captured by the traditional six-level hierarchy.

Non-equilibrium dynamics: Importantly, the framework does not presuppose whether transmembrane processes operate under steady-state or non-equilibrium conditions; rather, it accommodates both, depending on the spatial and temporal scope of analysis. Many physiological processes are locally non-equilibrium in nature yet collectively contribute to relatively stable system-level conditions. For example, gas exchange across alveolar–capillary and tissue interfaces involves continuous local gradients, but these distributed processes together support stable arterial levels of O_2_, CO_2_, and blood pH. From this perspective, local non-equilibrium mechanisms can be interpreted within a coordinated system-wide context. At the same time, the impact of such processes depends on their scale and magnitude. For instance, although action potentials involve transient ionic fluxes, the total ionic movement is small relative to the overall intracellular ionic content and does not significantly perturb the global cellular environment.

Visual representation: Equally important is the *visual expressiveness* of the framework. The color of the ECF—healthy blue in the osmoregulation example, gray in hypoxic responses, and brown and clouded in metabolic overload—provides an immediate, system-wide impression of the state of the internal environment. Because every intracellular domain is drawn inside the same ECF, a simple color shift conveys that the entire organism is experiencing a changed condition, while different cell types “read” and respond to that condition in distinct ways. This visual representation reinforces the relational logic of the framework and makes whole-body coordination, regulatory response, or failure visible at a glance.

Scalability across levels of abstraction: The framework is inherently scalable. Any intracellular domains depicted in these examples can be further expanded (zoomed in) to reveal underlying cellular and subcellular mechanisms without altering the relational logic of the framework. Conversely, multiple intracellular domains can be simplified or grouped (zoomed out) to provide a more abstract, system-level view. This scalability is not limited to intracellular detail. More broadly, the framework permits the representation of anatomical structures across multiple levels of biological organization from subcellular components to cells, tissues (e.g., the hypothalamus and posterior pituitary in **Figure 2**, and the diaphragm and bones in **Figure 3**), organs (e.g., the lungs and kidneys in **Figure 3**), and organ systems when such detail is relevant. Rather than prescribing a fixed structural resolution, the framework allows structural detail to be introduced or simplified as needed, while preserving the same underlying relational logic.

Conceptual coherence across abstraction levels: At a macroscopic level, physiology teaching often treats the ICF as a unified compartment, reflecting the broadly similar composition of intracellular environments across cell types. At more detailed levels, however, functionally important differences between cell types become evident. The 5-domain framework accommodates both perspectives. At its highest level of abstraction, it represents the ICF as a unified domain (**Figure 1****),** whereas in applied contexts this abstraction can be refined to distinguish cell-type-specific intracellular domains and their associated functional properties (**Figures 2**–**4**). Importantly, this flexibility is not arbitrary but arises from the preservation of the same relational organization across levels of abstraction, allowing the framework to remain conceptually coherent while transitioning between integrative and mechanistic perspectives.

Implications for integrative physiology: Together, these features highlight a key strength of the framework: its ability to support both system-level integration and localized mechanistic representation within a unified organization. This makes it particularly well-suited for studying coordinated system behavior and emergent phenomena, thereby supporting the development of integrative physiology. The three examples in this section illustrate one aspect of this potential, namely, the organization of distributed biological events into a coherent relational representation. In the next section, the same domain logic is extended to organize quantitative relationships, forming an organizing tool termed the blueprint for integrating quantitative functions across biological systems.

## 4 Extending the 5-domain framework: a blueprint for quantitative functional organization

While the first three examples illustrates how the framework organizes physiological and pathophysiological processes, the same relational logic can be extended to organize quantitative relationships in physiology and other life sciences. This was, in fact, where the entire journey began. My initial intention was simply to find a systematic way to arrange the many curves and equations that appear across disciplines. However, the conventional 6-level structural hierarchy provided no functional logic for such an organization. Many quantitative relationships do not belong to a single anatomical level, several clearly span multiple levels, and—most critically—the hierarchy has no place for transmembrane processes, even though these processes underlie many well-known equations such as membrane-potential relationships, ion-channel currents, and transporter kinetics.

The need to place mathematical relationships into a meaningful functional order ultimately compelled me to work out an alternative functional logic: the 5-domain functional framework. With this framework established, the mathematical relationships began to fall naturally into place in a relational, hierarchical space. **Table 1** organizes the sample mathematical relationships with the rows representing the first four functional domains and the columns categorizing the types of mathematical relationships. Since the independent variable of many curves is time, the position of each relationship in the table is generally determined by the functional domain in which the dependent variable operates or is measured. The organization is not rigid and does not need to be; its value is that it provides an expandable blueprint of the functional order of higher-level quantitative relationships across different layers of structures, scales, and disciplines.

**Table 1:** Blueprint for quantitative functional organization (BQFO) derived from the 5-domain functional framework with the pan-molecular domain (Domain ❺) functioning implicitly or explicitly across all domains.

Functional Domain	Mathematical representations of biological processes				
	Exponential	Logarithmic	Sigmoidal	Hyperbolic	Linear
Organism Functional Sphere (Domain ❶) and Beyond	• Ebbinghaus’s Forgetting Curve (↓) (Murre and Dros, 2015) • Age-performance relationship (↑, then ↓) (Berthelot et al., 2012) • Early embryo growth (cleavage, ↑)	• Stimulus-perception relationship (↑) (Portugal and Svaiter, 2011) • Sensory adaptation (↓) (Xu et al., 1996)	• Bacteria and insect growth (↑) (Cao et al., 2019) • Human growth in height (↑) (Thompson, 2022) • Learning curve (Reves, 2000) or skill acquisition (↑) (Nitsche et al., 2019)	• Growth of the human population (Golosovsky, 2010) • Sensory stimulus strength-reaction time relationship (↓) (Medina, 2012)	• Cardiac output vs. whole body O_2_ intake during moderate, steady-state exercise (Beck et al., 2006) • Age-related height decline(Sorkin et al., 1999)
Extracellular Functional Domain ❷ (Organ System, Organ, Tissue)	• Activation of Factor VII during blood clotting (↑) (Shibeko et al., 2010) • Viral load increase (↑) (Challenger et al., 2022) • First-order plasma drug clearance (↓) (Baca and Golan, 2017) • Prostate cancer cell growth (↑) (Blagoev et al., 2021)	• pH scale • Henderson-Hasselbalch Equation	• O_2_-Hb dissociation curve (↑) • Dose-response curve of inhibitory drugs (reverse S-shape, ↓) • Filterability vs. neutral dextran size (↓)	• Frank-Starling curve (hyperbolic-like, ↑) • Alveolar ventilation equation (↓) • Plasma creatinine and creatinine clearance (↓) • Boyle’s law (↓) in the lung	• Lactate increase during moderate exercise (↑) (Binder et al., 2008) • Total CO_2_ content vs. CO_2_ partial pressure in arterial blood • Urinary excretion of NH_4_^+^ vs. urinary pH (↓)
Transmembrane Functional Domain ❸	• Rising (↑) and falling (↓) of a graded potential • Recovery of voltage-gated Na^+^ channels (↑)	• Nernst equation	• SGLT1 transport (↑) (Subramaniam et al., 2019) • Voltage-gated K^+^ channel current vs. membrane potential (↑) (Peng et al., 2017)	• Glucose transport via GLUT1 (↑) (Custódio et al., 2021) • Plasma membrane Ca^2+^-ATPase kinetics (↑) (Filomatori and Rega, 2003)	• Fick’s law (↑) • Van ‘t Hoff’s law under constant temperature • Ohm’s law involving membrane conductance
Intracellular Functional Domain ❹ (Extranuclear, Transnuclear membrane, Intranuclear subdomains)	• Viral replication with unlimited resources (↑) (Regoes et al., 2005) • Protein degradation (first-order kinetics, ↓) (Alber and Suter, 2019) • mRNA transcription in certain conditions (↑) (Deneke et al., 2012)	• pH scale • Henderson-Hasselbalch Equation	• GPCR-induced cAMP generation (↑) (Trehan et al., 2014) • Kinetics of allostatic enzymes (Traut, 2008) • *lac* responses vs. time (↑) (Michel, 2013)	• Enzyme kinetics: Michaelis-Menten equation (↑) (Voet and Voet, 2004) • Sarcoplasmic Ca^2+^-ATPase activity vs. [ATP] (↑) (Rivera-Morán and Sampedro, 2023)	• Growth of actin filaments (polymerization phase, ↑) (Vavylonis et al., 2005) • Telomere shortening (age-related, ↓) (Vaiserman and Krasnienkov, 2021)

The quantitative relationships presented in this table were selected from multiple disciplines, including physiology, biochemistry, pharmacology, immunology, microbiology, cell biology, neuroscience, exercise physiology, population biology, and related fields, to illustrate the interdisciplinary scope of the BQFO. Relationships without accompanying citations represent classical physiological relationships that are widely available in standard physiology textbooks.

I was surprised that the 5-domain framework, which was originally motivated by a practical organizational need, turned out to have much broader explanatory power. The same functional logic that helped arrange the equations in **Table 1** also helped integrate the physiological, allostatic, and pathophysiological processes shown in the first three examples. In retrospect, the encapsulating relationship between ECF and ICF is not merely a conceptual abstraction but a methodological turning point that allows the transmembrane domain to emerge as a necessary link in the causal chain. With this link in place, the entire physiological “game board” becomes alive and functional, as events inside the ICF and events in the ECF can now be systematically connected through Domain ❸.

The organization in **Table 1** is only a starting blueprint. It can be expanded to include more curves, more systems, and more interdisciplinary connections. The key point is that the 5-domain framework can serve as a higher-order epistemological and operational tool that provides an integrative way of viewing and organizing functions that is not restricted by anatomical layers. Its dual capacity to organize both biological events and quantitative relationships suggests that the framework may help support future developments in integrative physiology and interdisciplinary teaching and research.

Together, these four examples illustrate the explanatory power and practical usefulness of the 5-domain functional framework. By showing how distributed cell types interact within a shared extracellular environment and their specific transmembrane mechanisms, the framework provides a coherent way to connect intracellular processes with whole-body outcomes as well as a functional logic capable of organizing both physiological phenomena and mathematical relationships. This combination of integrative scope and organizational flexibility is what gives the framework its conceptual versatility and methodological promise, laying the groundwork for the broader implications discussed in the next section.

## 5 Significance and implications of the 5-domain functional framework

Epistemologically, the heart of the 5-domain functional framework is the simple idea that the ECF envelops the ICF, followed by the emergence of the transmembrane domain that links the two compartments. This framework appears a suitable epistemological tool that highlights the unity and inseparability of bodily functions and may be especially useful in integrative physiology, fields in which bodily functions cannot be understood in isolation.

Methodologically, the 5-domain framework is highly practical, operational, convenient, and flexible as shown in the four examples above. It allows readers to see instantly which intracellular domains are sensitive to a particular disturbance or change in the internal environment and how differently they respond depending on their specific transmembrane mechanisms and intracellular machinery. The framework is also scalable. One can zoom in to illustrate specific transporters or signaling pathways or zoom out to show broader causal flows across domains. This makes it adaptable to both research and teaching, where different levels of abstraction may be required. Importantly, this scalability is grounded in the preservation of the same underlying relational logic across levels of abstraction, allowing the framework to maintain conceptual coherence while transitioning between system-level integration and localized mechanistic detail. Its power to organize both coordinated bodily functional processes and quantitative relationships makes it a powerful approach to integrative physiology and interdisciplinary research and education.

In research, the 5-domain framework may offer a shared functional language that can bridge disciplines. Physiologists, neuroscientists, immunologists, pharmacologists, and molecular biologists often approach their work from different “levels,” but all of their findings may be mapped into the same domain logic. This supports interdisciplinary communication and helps researchers see how local mechanisms participate in global functions, while also offering a way to explore how global physiological states may constrain and shape the behavior of subsystems. Because all intracellular domains are nested within a shared extracellular environment, systemic parameters can be represented as common boundary conditions that simultaneously influence multiple cell types.

In education, the framework will help students place isolated mechanisms into a relational context instead of memorizing disconnected processes, making homeostasis, allostasis, and pathophysiology easier to understand.

The framework makes complex functions more approachable and creates opportunities for further conceptual development. Specifically, it is possible and feasible to graphically illustrate a wide range of bodily functions in the unified 5-domain functional framework in an atlas that would provide foundational templates for both teaching and research. Instructors and investigators could select a template and adapt it, allowing a shared visual logic to support communication and understanding across topics. A handbook could systematically collect mathematical relationships and organize them by the 5-domain functional framework for a systematic view of quantitative physiology or biology. This handbook could become a useful tool across disciplines, offering teachers, students and researchers a functional map of equations that are now scattered across textbooks.

The expandable BQFO (**Table 1**) also supports user-driven exploration. A user may choose any set of relationships, place them into the table according to the domain logic, and then study how a specific change, such as an altered ECF environment, would influence the dependent variables across the selected relationships. This creates an immediate global overview of coordinated shifts and can reveal connections that are invisible when equations are learned one at a time.

For example, during acclimatization to high-altitude hypoxia, a user could simultaneously examine the oxygen–hemoglobin dissociation curve, the alveolar ventilation equation, the relationship between cardiac output and whole-body oxygen intake, Fick’s law of diffusion, and changes in mRNA transcription under hypoxic conditions. Placing these relationships within the same functional-domain logic allows coordinated alterations across organismal, extracellular, transmembrane, and intracellular domains to be viewed together rather than as isolated phenomena. Such an arrangement may help reveal how changes in the internal environment propagate through multiple levels of functional organization.

The example above still reflects a relatively familiar and topic-centered way of selecting relationships. More systematic exploration may become possible by starting with a defined perturbation (e.g., high-altitude hypoxia, infection, metabolic overload, tumor development, or inflammation) and then examining coordinated relational changes across all functional domains. Such an approach may help reveal broader system-level patterns that are difficult to recognize when relationships are studied separately within traditional disciplinary boundaries.

In sum, the 5-domain framework is not only conceptual but operational: a flexible framework organized around functional relationships for examining complex functional systems.

## 6 Conclusions

A broadly usable 5-domain functional framework will help transform fragmented findings in integrative physiology into a coherent understanding. The framework introduced here represents an initial attempt in this direction. Through examples ranging from homeostasis and allostasis to maladaptation and quantitative relationships, the framework demonstrates both conceptual clarity and practical flexibility. It allows complex functions to be represented and understood within a unified relational logic that can be expanded or simplified as needed. Importantly, this flexibility is not arbitrary but arises from the preservation of the same underlying relational logic across levels of abstraction, which enables the framework to maintain coherence while transitioning between integrative and mechanistic perspectives. Within this relational organization, the framework can support both bottom-up investigations of how local mechanisms contribute to system-level emergent functions and top-down analyses of how global physiological or pathophysiological states constrain and shape the behavior of subsystems. For these fields, the framework offers both an epistemological perspective and an operational method that form a workable tool for teaching, research, and interdisciplinary communication. It also points toward new possibilities, such as a visual atlas of integrative physiology or an organized handbook of mathematical relationships.

Building on this foundation, the Blueprint for Quantitative Functional Organization (BQFO) provides a reference for systematic and large-scale functional studies. As the BQFO expands, the mathematical relationships it can accommodate become essentially limitless. Bringing them together in one functional space opens the possibility of discovering deeper connections across equations that have never been examined together. Such a platform may allow functional research to move into a broader and more unified landscape, where system-level patterns and principles can begin to emerge in ways that were previously inaccessible.
